# The Role of Notch in the Cardiovascular System: Potential Adverse Effects of Investigational Notch Inhibitors

**DOI:** 10.3389/fonc.2014.00384

**Published:** 2015-01-13

**Authors:** Paola Rizzo, Donato Mele, Cristiana Caliceti, Micaela Pannella, Cinzia Fortini, Anthony George Clementz, Marco Bruno Morelli, Giorgio Aquila, Pietro Ameri, Roberto Ferrari

**Affiliations:** ^1^Department of Medical Sciences, University of Ferrara, Ferrara, Italy; ^2^Laboratory for Technologies of Advanced Therapies (LTTA), University of Ferrara, Ferrara, Italy; ^3^GVM Hospitals, Cotignola, Italy; ^4^Azienda Ospedaliero-Universitaria di Ferrara, Cona, Italy; ^5^Department of Chemistry, DePaul University, Chicago, IL, USA; ^6^Research Center of Cardiovascular Biology, Department of Internal Medicine, University of Genova, Genova, Italy

**Keywords:** Notch inhibitors, cardiac remodeling, cardiotoxicity, endothelial dysfunctions, cancer therapy

## Abstract

Targeting the Notch pathway is a new promising therapeutic approach for cancer patients. Inhibition of Notch is effective in the oncology setting because it causes a reduction of highly proliferative tumor cells and it inhibits survival of cancer stem cells, which are considered responsible for tumor recurrence and metastasis. Additionally, since Delta-like ligand 4 (Dll4)-activated Notch signaling is a major modulator of angiogenesis, anti-Dll4 agents are being investigated to reduce vascularization of the tumor. Notch plays a major role in the heart during the development and, after birth, in response to cardiac damage. Therefore, agents used to inhibit Notch in the tumors (gamma secretase inhibitors and anti-Dll4 agents) could potentially affect myocardial repair. The past experience with trastuzumab and other tyrosine kinase inhibitors used for cancer therapy demonstrates that the possible cardiotoxicity of agents targeting shared pathways between cancer and heart and the vasculature should be considered. To date, Notch inhibition in cancer patients has resulted only in mild gastrointestinal toxicity. Little is known about the potential long-term cardiotoxicity associated to Notch inhibition in cancer patients. In this review, we will focus on mechanisms through which inhibition of Notch signaling could lead to cardiomyocytes and endothelial dysfunctions. These adverse effects could contrast with the benefits of therapeutic responses in cancer cells during times of increased cardiac stress and/or in the presence of cardiovascular risk factor.

## Introduction

The development of new therapeutic strategies for many types of cancers has prolonged the cancer-free survival time of an increasing number of patients. Since chemotherapeutic agents, radiation therapy, and biological agents all have the potential to injure the cardiovascular system, it is not surprising that cardiotoxicity has revealed to be an important side effect of many oncology drugs and, depending on the drug type, between 5 and 25% of cancer patients develop some type of cardiac dysfunctions (1–3). The definition of cardiotoxicity comes from the Cardiac Review and Evaluation Committee (CREC), a retrospective study that sought to estimate the cardiotoxicity of trastuzumab, a receptor tyrosine kinase inhibitor (4). The CREC defined cardiotoxicity the development of (1) cardiomyopathy characterized by a decrease in left ventricle ejection fraction (LVEF) that was either global or more severe in the septum; (2) symptoms of congestive heart failure (CHF); (3) associated signs of CHF, including but not limited to S3 gallop, tachycardia, or both; and (4) decline in LVEF of at least 5 to <55% with accompanying signs or symptoms of CHF, or a decline in LVEF of at least 10% to below 55% without accompanying signs or symptoms. Any one of the four criteria was sufficient to confirm a diagnosis of cancer drug-induced cardiotoxicity. It is important to point out that this definition does not include subclinical cardiovascular damage and it does not take in consideration toxicity, which appears long after the treatment has been interrupted (1). Furthermore, cardiotoxicity can also manifest as valvular disease, pericarditis and myocarditis, or as an effect on the vasculature (hypertension, alteration of coagulation cascade, endothelial cells damage) that could indirectly affect heart function (1).

Early detection of cardiotoxicity from oncologic treatment is crucial to design a strategy to limit cardiotoxic effects. Decreased LVEF is well established as a strong predictor of cardiac morbidity and mortality in general. Assessment of LVEF is used to prevent irreversible cardiac damage and heart failure; however, LVEF is not sensitive enough to reveal a subclinical myocardial dysfunction, which can lead to symptomatic CHF and death (5). The dissection of the molecular mechanisms underlying heart dysfunction caused by cancer treatment could help to identify biomarkers for early detection of cardiotoxicity and could lead to the development of new therapeutic strategies able to interfere with onset and progression of cancer drug-related cardiac dysfunction.

Cancer continues to be a moving target and the challenge is to identify new molecular targets to overcome the unsolved issue of resistance to treatment. The Notch pathway is upregulated in the majority of cancers where it makes cancer cells resistant to apoptosis-causing agents. Therefore, Notch inhibition is being investigated for cancer therapy. In this review, we will first provide an overview of the clinical relevant cardiovascular side effects and of the molecular mechanism of cardiotoxicity associated with some of commonly used cancer treatment. We will then highlight the biological processes regulated by Notch in the cardiovascular system to discuss the possibility that these investigational Notch inhibitors could cause cardiotoxicity.

## Cardiotoxicity Associated with Common Agents Used for Cancer Therapy

The introduction of anthracyclines (doxorubicin, daunorubicin, or epirubicin) as chemotherapic agents has added a very effective tool to cancer therapy. The clinical chemotherapeutic use of doxorubicin is limited by cardiotoxicity which in the absence of other risk factors is tolerated up to a cumulative dose of 300 mg/m^2^ with a rate of HF of <2% (3). Above this dosage, the rate increases exponentially and a study conducted on 630 patients has shown that an estimated 26% of patients would experience doxorubicin-related CHF at a cumulative dose of 500 mg/m^2^ (6). These dosages refer to patients that were <65 years old and in the absence of other factors that seem to influence the toxicity such as genetic predisposition, arterial hypertension, and combination with other anticancer agents (3).

Owing to these dramatic cardiotoxic effects, high doses are no longer used but subacute and chronic cardiac effects of anthracycline are still a problem (7). The clinical assessment of the myocardial damage caused by anthracyclines is difficult since more than 50% of patients that will develop HF show <30% reduction of LVEF (6). To identify abnormalities in breast cancer survivors 1 year after treatment with anthracycline, 2D myocardial strain (rate) imaging is more sensitive than conventional echocardiography (8). Three-dimensional echocardiography (RT-3DE) is also more effective in detecting abnormalities in cardiac function in long-term survivors of childhood cancer after cardiotoxic therapy (9). In terms of biomarkers, levels of NT-proBNP (N-terminal of the pro-hormone brain natriuretic peptide) and prolongation of QT-interval (which measures the electrical depolarization and repolarization of the ventricles) are useful markers for course-to-course evaluation of anthracycline-induced cardiotoxicity (10).

The mechanism of anthracycline-induced toxicity is complex and not fully understood. Anthracyclines cause the formation of reactive oxygen species within cardiac cells, partly by reacting with intracellular free iron. It has long been postulated that anthracycline-induced oxidative stress initiates a cascade of alterations eventually leading to cardiomyocyte damage and death. However, recent evidence suggests that the primary event in the pathogenesis of anthracycline cardiotoxicity is the inhibition of topoisomerase II activity (11). Consistent with this hypothesis, a randomized study performed in breast cancer patients to investigate whether free radical scavenger super oxide dismutase (SOD) would protect against anthracycline-mediated cardiotoxicity gave negative results (12). Regardless of the molecular event at the origin of anthracycline cardiotoxicity, this latter then develops through the impairment of many cardiac cell functions, such as decreased expression of key proteins, disruption of Ca2+ homeostasis, induction of mitochondrial DNA lesions and perturbation of mitochondrial bioenergetics, degradation of myofilamental and cytoskeletal proteins, and interference with various pro-survival kinases [for an extensive review of these alternative mechanisms the reader is referred to Simunek et al. (13)]. Of note, anthracycline toxicity involves not only the population of terminally differentiated cardiomyocytes but also the pool of cardiac progenitor cells (CPCs). These are c-kit, stem cell antigen 1 (Sca1), and multi-drug resistant gene product 1 (MDR1) positive, self-renewing, and multi-potent cells that play a role in cardiac repair (14–17). Therefore, anthracycline depletion of CPCs may hinder the capability of cardiac tissue to regenerate following minor injuries (18, 19). The loss of cardiomyocytes is accompanied by interstitial fibrosis (20); at present, it is not known whether this is purely reactive or it is also the consequence of the direct effect of anthracyclines on fibroblasts.

Trastuzumab is a humanized monoclonal antibody that interferes with human epidermal growth factor receptor 2 (HER2), a member of the epidermal growth factor receptor family involved in modulation of cell proliferation and survival, which is overexpressed in 25–30% of all breast cancers (21). Trastuzumab treatment causes HF and asymptomatic decline in systolic function in around 25% of patient when administered sequentially or in combination with anthracyclines (22). In a mouse model of cardiotoxicity that recapitulates the clinical therapeutic protocols of consecutive cycles of doxorubicin followed by trastuzumab, a detrimental synergistic global cardiac injury extending to both the LV and RV chambers was observed (23). Cardiomyocytes express HER2, which activates survival pathways, in response to stressor agents. It is thought that inactivation of HER2 by trastuzumab in cardiomyocytes impairs their ability to activate reparative pathways following anthracycline-induced damage (20). The use of trastuzumab alone or in combination with paclitaxel, a first line generic cytoskeletal drug, is also associated with cardiotoxicity, even though in a lower number of patients (24). Remarkably, the mechanism by which trastuzumab monotherapy damages cardiomyocytes remains rather unclear, since in the absence of noxious stimulus the activity of HER2, if any, may be very low (25). Furthermore, the cardiac dysfunction rate of novel HER2-targeted therapies (lapatinib, pertuzumanb, T-DM1, neratininf, and afatinib) is significantly lower than that related to trastuzumab (26). In any case, and differently from anthracyclines, trastuzumab-induced cardiotoxicity has been so far considered reversible if treatment is interrupted (27) and no structural changes have been identified in cardiomyocytes following trastuzumab treatment (28). Newer reports are challenging this view and show changes in myocardial genes essential for DNA repair, cardiac and mitochondrial functions associated with impaired LV performance in mice (29). Recently, it has been reported that trastuzumab may exert adverse effects also on the coronary and peripheral vasculature (30). In order to limit cardiac damages, newer protocols recommend 1 year of trastuzumab therapy in patients with HER2-positive breast cancer ≥1.0 cm in size; even after 1 year, treatment still is interrupted due to cardiotoxicity in 13.6% of patients (31).

Interference with tumor angiogenesis is a promising avenue for cancer therapy (32). Vascular endothelial growth factor (VEGF) targeting by antibodies (bevacizumab) or by small-molecule tyrosine kinase inhibitor (sunitinib, sorafenib) has become an option for treatment of patients with a variety of solid tumors. Similarly to trastuzumab, bevacizumab causes heart dysfunction in 3.8% of patients (3). Cardiotoxicity has been reported for 4.1 and 1% of sunitinib- and sorafenib-treated patients, respectively. The molecular mechanism of cardiotoxicity of these drugs is still unclear. No ultrastructural changes in cardiomyocytes have been observed and since they cause arterial hypertension, it has been suggested that heart dysfunction could be a secondary effect (3).

## Notch Inhibition for Cancer Therapy

The Notch pathway is a fundamental signaling system involved in making decision on “cell fate” (33). Mammals have four Notch receptors (Notch 1–4) and five ligands (Delta-like-1, -3, -4 and Jagged-1 -2) both located on the cell surface and involved in the communication of adjacent cells.

Notch proteins display a selective cellular and tissue distribution. Notch1 is broadly expressed in diverse cell types, whereas Notch 4 is preferentially expressed in the endothelium (34, 35). Delta-like ligand 4 (Dll4) was primarily described as endothelial-restricted molecule (36) but its expression has been recently reported in a wider number of tissues (37). Notch receptors are synthesized as single-chain precursors and cleaved into an extracellular and a transmembrane subunit in the Golgi apparatus. These two subunits are held together on the cell membrane by non-covalent bonds. Binding of ligand triggers the removal of the extracellular subunit by a disintegrin and metalloprotease (ADAM) followed by an intramembranous cleavage by γ-secretase, a multisubunits membrane protease. This last cut by γ-secretase releases the active form of Notch intracellular (NIC), which translocates into the nucleus where it displaces corepressors and activates coactivators that modulate transcription via the recombinant signal binding protein 1 for Jκ (RBP-Jκ) transcription factor (38). The most well-known Notch target genes belong to the Hes and Hey gene families, which are negative regulators of transcription but recent work has revealed that the number of target genes is even higher than once thought (33). Notch activity is tightly regulated by post-translational modifications such as phosphorylation, glycosylation, ubiquitination-mediated degradation (39), and by cross-talks with other key proteins including the inflammatory cytokines tumor necrosis factor α (TNFα) and interleukin 1β (IL1β) (40), the nuclear factor-kappa-light-chain-enhancer of activated B-cell (NF-κB), the PEA3 family of transcription factors (41, 42), the estrogen (43), and the vascular epidermal growth factor (VEGF) receptors (44). As a result, the consequence of Notch activation is exquisitely cell-context dependent and the output very difficult to predict.

Beginning with the first reports of an involvement of Notch1 in the development of 10% of T-cells acute lymphoblastic leukemias (T-ALL), investigations conducted in the last 20 years have shown Notch activation in the majority of solid tumors and hematological malignancies (45). Activation of Notch signaling *in vitro* and *in vivo* increases cancer cell survival in the presence of commonly used chemotherapy agents (38). Active Notch signaling is needed for survival of cancer stem cells (46) and to sustain angiogenesis within the tumor environment (44).

The requirement of an active Notch signaling for cancer growth has generated high enthusiasm in the recent years about the possibility to target this pathway for cancer therapy. There are about 30 clinical trials ongoing to evaluate safety and efficacy of γ-secretase inhibitors (GSI), administered alone or in combination with standard care treatments (registered at www.clinicaltrials.com) in cancer patients. In order to minimize toxicity, more specific approaches are being developed such as targeted antibodies directed against individual Notch family members (47). Blocking Dll4, the Notch ligand specifically involved in modulation of angiogenesis, has given promising results in interfering with cancer growth: administration of anti-Dll4 agents in breast cancer xenografts promotes excessive sprouting, which leads to unproductive angiogenesis (48).

## Role of Notch in Cardiovascular System

While the role of Notch receptors and ligands in vasculogenesis during the development is well established, we are just beginning to understand the complex and multiple roles played by this pathway in post-natal vasculature. Notch receptors 1, 2, 3, and 4 and Delta-like ligands 1, 4 and Jagged 1, 2 ligands are expressed in the adult vasculature (49). Notch1 and Notch4 are predominantly endothelial, prominent in both arteries and veins, while the expression of Notch2 is confined to the pulmonary endothelium and Notch3 is primarily expressed in adult arterial vascular smooth muscle cells (VSMCs) in large conduit, pulmonary, and systemic resistance arteries (50, 51). Notch plays a major role in the modulation of angiogenesis and therefore this aspect of the Notch signaling has been object of intensive investigation during the last 15 years due to the importance of angiogenesis for tumor growth (52). Notch is activated in the context of vascular injury, suggesting an important role for this pathway also in limiting damages to the vascular structure (49).

Notch1 and Jagged1 play a pivotal role in organogenesis of the heart (53). In the post-natal heart, Notch signaling is absent under physiological condition but its reactivation in the overloaded or damaged myocardium suggests a role in the biological processes involved in heart repair (15, 54–58).

### Role of notch in endothelial dysfunctions

The endothelium controls vascular functions such as vasomotion, thrombosis, platelet aggregation, and inflammation. Endothelial dysfunction (ECD) is a broad term that includes not only denudation caused by apoptosis of endothelial cells and by inability to replace desquamating cells but also reduced synthesis of molecules with a protective effect on the vasculature (i.e., nitric oxide) and the expression of proteins, such as intercellular adhesion molecule-1 (ICAM-1) and vascular cell adhesion molecule-1 (VCAM-1), which mediate the adhesions of inflammatory cells on the surface of endothelium (endothelium activation) (59). ECD induced by inflammatory conditions is not only the first step toward the formation of atherosclerotic plaques (60, 61) but is also thought to be involved the progression of cardiac disease (62). Consistently, epidemiological studies have shown an association between systemic inflammation and poor prognosis in patients with cardiovascular diseases (63–65) and human umbilical veins endothelial cells (HUVECs) cultivated in the presence of serum from HF and acute myocardial infarction (AMI) patients show increased levels of apoptosis (66, 67), a critical marker of ECD.

Notch plays an important role in protecting endothelial cells from apoptosis induced by conditions such as inflammation, oscillatory blood flow, and ischemia. *In vitro* and *in vivo* treatment with inflammatory cytokines TNFα and IL1β leads to dysregulation of Notch signaling (down-regulation of Notch4 and induction of Notch2), endothelial cells activation (ICAM-1 and VCAM-1 production), and apoptosis (40, 68). A prominent role, in particular, for Notch4 in the protection of endothelial cell has been shown in cardiac allograft vessels in which impaired Notch4 expression caused by pro-inflammatory cytokines promotes endothelial cells dysfunction and transplant arteriosclerosis (69). Exposure of microvascular endothelial cells to high laminar blood flow conditions (which induces a protective gene expression profile in the endothelium) results in upregulation of Notch1 mRNA, which enhances cells survival by upregulating the anti-apoptotic proteins Bcl2 (70). Under ischemic conditions, VEGF-A promotes not only migration and proliferation but also protection endothelial cells from apoptosis. Experiments in cultures of HUVECs grown in absence of serum to mimic an ischemic environment have shown that VEGF-A treatment is unable to protect cells from serum deprivation-induced apoptosis in absence of a functional Notch 1 signaling (71).

Notch also modulates endothelial cell proliferation in a complex way. When endothelial cells reach confluence, Notch1 is activated that in turn leads to p21Cip1 down-regulation and to cell cycle arrest, suggesting a role for active Notch1 in contact inhibition of the endothelial monolayer (72). On the other hand, it has been shown that Notch inhibition in HUVECs leads to increased intracellular ROS formation and inhibition of cells proliferation (73) and cathepsin K induces endothelial cells proliferation *in vivo* by activating Notch1 (74). More recently, Schoeber et al. have shown that shear stress-induced down-regulation of miR126-5p leads to upregulation of DLK1 which, by inhibiting Notch1-Hes5 signaling, prevents endothelial cells proliferation and endothelium repair in the athero-prone regions of the aortic arch (75).

Nitric oxide (NO) production is the main indicator a functional endothelium. NO diffuses from the endothelium into the adjacent smooth muscle where it activates guanylate cyclase, which in turn induces cGMP-mediated reduction of contraction of smooth muscle cells and maintains basal vasodilator tone. NO is also involved in preventing platelet and leukocyte activation and adhesion to the vessel wall (76). Notch1 in the tumor vasculature is involved in NO production by a VEGF-mediated regulation of eNOS (77). Furthermore, in bone marrow (BM)-derived endothelial cells, Notch1 binds to the promoter and inhibits the synthesis of miR155 (78), a pro-inflammatory miRNA downstream of NF-κB, involved in down-regulation of eNOS mRNA (79).

### Role of notch in ischemic tissues

Angiogenesis is critical for the reperfusion of ischemic tissues. As previously discussed, Dll4/Notch1-mediated signaling modulates VEGF-A-driven angiogenesis by regulating the number of sprouts (new branches) on endothelial cells. According to a widely recognized model, the interplay between Dll4/Notch1/VEGFR determines the balance between the number of tip cells (leading and guiding the blood vessel sprout) and stalk cells (proliferating cells forming the vascular lumen,) (80). Specifically, the tip cell expresses Dll4 and has little Notch activity. Dll4 signals through Notch1 in the adjacent stalk cells and limits sprouting by reducing the response to VEGF-A through the down-regulation of VEGFR-2 (81) and the upregulation of VEGFR-1, which functions as a decoy receptor that sequesters VEGF-A (82). This model is now been revised and it assumes that tip and stalks cells are not static but that can exchange roles and that this rearrangement is mediated by differential dynamics of VE-cadherin junctions regulated by Notch/VEGFR signaling (83). Recent published work has shown that Notch-dependent VEGFR3 upregulation allows angiogenesis in absence of VEGF–VEGFR2 signaling (84).

The importance of the Dll4/Notch1 role in angiogenesis has been recently further confirmed by studies in zebrafish in which blood flow-mediated suppression of Dll4/Notch signaling is required to promote angiogenesis in response to hypoxic signaling (85). The molecular effectors of Notch in this context are not completely defined. Two newly identified targets of Notch-mediated angiogenenesis are Sox17 in retina (86) and eNOS in the tumor vasculature (77).

Arteriogenesis is the maturation of arterio-arteriolar anastomoses by the recruitment and coating of pre-formed vessels with pericytes or VSMCs. Pericytes are among the first cells to invade newly vascularized tissues and locate at the growing front of the endothelial sprouts by determining the location of sprout formation and by guiding newly formed vessels and Notch activity is required for their proliferation and to mediate pericyte–endothelial interaction (87). Notch inhibition disturbs vessel stability and leads to pericytes detachment followed by extravasation of mononuclear cells (88). Furthermore, changes in hemodynamic forces caused by the occlusion of an artery promote activation of Notch and of NF-κB, which are both necessary for arteriogenesis of the ischemic limb (89).

Endothelial progenitor cells (EPCs) contribute to re-endothelization and play a role in the neo-vascularization of ischemic tissues and in tissue repair. Consistently, it has been reported that the number of circulating EPCs increases in patients with cardiovascular disease (90) and in diabetic patients with diabetic foot syndrome caused by impaired angiogenesis the number of EPC is reduced (91). Jagged1-derived Notch signals from the BM microenvironment are critical for EPC-mediated vasculogenesis (92). Notch-RBP-Jk signaling regulates the mobilization and function of EPCs by modulating the expression of CXCR4, the receptor for stromal derived factor 1 (SDF-1) involved in EPCs chemotaxis (93). In hypercholesterolemic mice, Notch1 signaling regulates EPCs activity during recovery from arterial injury: of interest low levels of cholesterol cause a mild inhibition of Notch, which enhances EPCs activity whereas high cholesterol levels strongly inhibit Notch causing EPCs apoptosis (94).

Consistently with the data shown so far which highlight the important role for Notch in angiogenesis and arteriogenesis, targeting Notch has proven to be effective in promoting wound healing and reperfusion of ischemic limbs (95–97).

### Role of notch in vascular muscle cells

Notch3, Jagged1, and Hes1 are expressed in VSMCs, the main cell type in the arterial wall, which plays a critical role in maintaining vascular structure and function (98). Age-associated intimal and medial thickening has been linked to reduced Jagged1-mediated Notch signaling (99). Furthermore, Notch signaling is downregulated in medial VSMCs of descending thoracic aortic aneurysm patients suggesting that impaired Notch signaling in VSMCs may contribute to the depletion of VSMCs that characterize this pathology (100). Down-regulation of Jagged1, Notch3, and Hesr1 in VSMCs by angiotensin II (AngII) has also been reported (98). AngII is a peptide hormone that causes vasoconstriction and subsequent hypertension, which, in turn, elicits structural modifications in small arteries and arterioles and reduction in lumen diameter (vascular remodeling). Since Notch3 inactivating mutations are present in cerebral autosomal-dominant arteriopathy with subcortical infarcts and leukoencephalopathy (CADASIL), an hereditary disease that causes stroke and dementia (101) and blockade of AngII generation helps to prevent stroke (102), it has been suggested that down-regulation of Notch could be part of the molecular mechanism by which AngII induces vascular complications (98). Other authors have shown that activation of Notch mediates the effect of AngII on vascular remodeling (103) and on abdominal aortic aneurism in ApoE knockout mice (104): more studies are needed to clarify the relationship between Notch and AngII pathways in vascular pathology.

Vascular smooth muscle cells are the main cellular component of atherosclerotic plaques and VSMCs apoptosis induces features of plaque instability in atherosclerosis (105). Experiments *in vitro* have shown that activation of canonical Notch1 and 3 signaling not only increases proliferation but also prevent apoptosis of VSMCs (106). It follows that sustained Notch inhibition in patients with atherosclerosis could have affect plaque stability and thrombus formation.

### Role of notch in myocardial repair

Pathological cardiac remodeling is defined as molecular, cellular, and interstitial changes that manifest clinically as changes in size, shape, and function of the heart after injury or stress stimulation (107, 108). It may occur not only after MI but also after pressure overload (aortic stenosis, hypertension), inflammation (myocarditis), idiopathic dilated cardiomyopathy, or volume overload (valvular regurgitation). After a MI, there is extensive myocyte necrosis and degradation of collagen fibers, which leads to infiltration of inflammatory cells for the re-absorption of necrotic tissue. The sliding of cardiomyocytes consequent to the degradation of the collagen fibers causes the thinning of the infarcted cardiac wall with ensuing regional dilatation. During this phase, fibroblasts deposit collagen on the thinned tissue in order to create a scar and limit further expansion of the focal dilatation. As a result, the geometry of the ventricle changes as it remodels: it becomes less elliptical and more spherical. Biopsies from HF patients show myocytes with a phenotype resembling fetal life with a pattern of embryonic myofilaments, down-regulation of sarcoplasmic reticulum calcium ATPase, increased expression of atrial natriuretic peptide and of ventricular myocytes expressing the i*f* current channels (109, 110). This series of events exert a beneficial effect on cardiac function at least for a limited period of time (111, 112).

**Figure 1:**
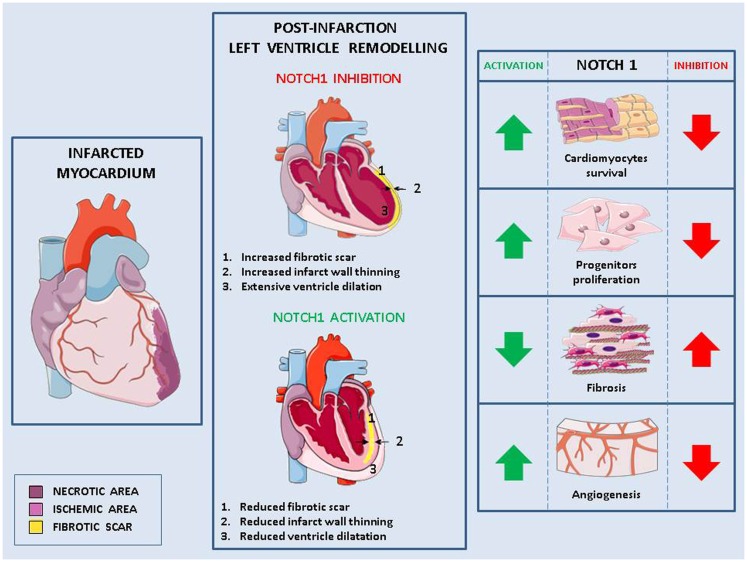
**Role of Notch in pathological remodeling**. Myocardial infarction causes cells injury (necrotic area) and the formation of an ischemic area in which cells are at risk of injury (left panel). This damage, exacerbated in absence of Notch signaling, leads to pathological left ventricle remodeling characterized by fibrotic scar, thinned myocardium wall, change of the ventricle shape and consequently, impaired cardiac function (middle panel). Activation of Notch1 in the infarcted myocardium reduces pathological remodeling by (1) increasing cardiomyocytes survival, (2) enhancing the proliferation of cardiac stem cells and favoring their differentiation into cardiomyocytes rather than fibroblasts, and (3) promoting angiogenesis (right panel).

Notch signaling is involved in crucial steps (cardiomyocytes survival and regeneration, fibrotic response, angiogenesis) determining both the extent of post-infarction myocardial damage and pathological LV remodeling (Figure 1).

Notch1 reactivation in the damaged myocardium has been linked to cardimomyocytes survival. In cardiomyocytes near the border with the infarct zone, increased Notch1 coincides with increased phosphorylation of the pro-survival protein Akt and reduced apoptosis (54). Similarly, there is decreased number of apoptotic cells following MI in mice overexpressing the active form of Notch1 in cardiomyocytes (113). Cardiomyocytes apoptosis caused by increased hemodynamic load in hypertensive mice is higher in absence of cardiac Notch1 signaling (15). Furthermore, in cardiomyocytes grown under hypoxia, Notch1 activation induced expression of anti-apoptotic genes (114) and inhibition of Notch signaling caused increased apoptosis (113).

In comparison to wild type, in mice overexpressing active Notch1 in cardiomyocytes, MI led also to increased number of ki67-positive cardiomyocytes, suggesting their re-entry in cell cycle and proliferation. However, no differences were found in this context in the number of phospho-Hist3 positive cardiomyocytes, suggesting that Notch activation induces incomplete cell cycle progression in adult cardiomyocytes (113). In agreement with these data, forced activation of Notch2 in mature cardiomyocytes led to cell cycle progression followed by G2/M interphase arrest block and apoptosis (115). These results suggest that following a myocardial damage, temporary activation of Notch1 would increase cardiomyocytes survival. It remains to be established whether, under these conditions, prolonged Notch activation would also be able to induce their proliferation.

The response to myocardial injury also includes the activation of CPCs (116). The ability of CPCs present in the adult myocardium to differentiate into cardiomyocytes in a post-infarction environment has been questioned by studies that have shown c-kit+ precursors support post infarction myogenesis in the neonatal, but not in the adult heart (117).

Notch is a fundamental pathway for proliferation and differentiation of resident CPCs. When they are actively proliferating, CPCs express high levels of active Notch1 (118). In contrast, Notch1 expression becomes undetectable when these cells lose their proliferative ability (119) indicating that active Notch1 signaling is required for the expansion of CPCs, but has to be downregulated to achieve terminal differentiation. CPCs express mainly Notch1 receptor (118). Its activation by Jagged1 on the surface of adjacent cardiomyocytes induces the expression of Nkx2.5, a transcription factor, which promotes proliferation and expression of cardiomyogenic transcripts, and inhibits the expression of markers of vascular cells (120). Thus, Notch1 favors myocyte lineage specification of CPCs and maintains them in a high proliferative state. By doing so, Notch1 exerts control not only of heart homeostasis but also of its adaptation to stresses and injuries: Notch1 inhibition in newborn healthy mice causes a 56% reduction of cardiomyocytes and induces dilated cardiomyopathy (118). Additionally, Notch1 inhibition causes a decrease of Nkx2.5 positive cells and a reduction in the generation of new myocytes in a mouse model of MI (120). In transgenic mice overexpressing Jagged1 on cardiomyocytes, remodeling caused by transaortic constriction is attenuated and there is improved cardiac function due to Notch1 activation in CPCs, which promotes their differentiation into Nkx2.5-positive cardiac precursor cells, rather than into fibrosis-causing myofibroblasts (56).

The number of BM-derived EPCs and mesenchymal stem cells (MSCs) is increased in the blood of patients with MI or HF (90, 121, 122). These cells participate to endothelial repair and neo-vascularization of ischemic organs, but they could be also involved in myocardium regeneration since they have been shown to differentiate *in vitro* to a cardiomyogenic phenotype (123). Experiments of co-colture of EPCs with Jagged1-expressing cardiomyocytes have shown that activation of Notch1 is necessary for the expression of cardiomyocytes markers in these cells (123). Additionally, deletion of Notch1 in BM-derived MSCs impairs their recruitment, proliferation, and survival leading to a decreased ability to repair the myocardium damage compared to MSC with a functional Notch1 signaling (124). Activation of Notch1 signaling in BM MSCs by soluble Jagged1 increases their differentiation rate into cardiomyocytes *in vitro* (125). Conversely, activation of Notch1 in immature cardiomyocytes by Jagged1 on MSCs enhances their proliferation (126).

The growth of new capillaries and arterioles is often inadequate in the post-infarction heart and this lack of adequate blood perfusion contributes to MI expansion and transition to HF (127). Notch1 is active in endothelial cells and VSMCs of cardiac vessels (113). In mouse heart, Notch1 activation by intramyocardial delivery of a monoclonal antibody (pseudo-ligand), 4 weeks after infarction, led to higher levels of angiogenesis markers, which were associated to reduced scar and improved cardiac functions (113). VEGF administration to chronically ischemic myocardium results in an upregulation of several Notch receptors and ligands and increased capillary and arteriolar density compared with ischemia alone (128). Similarly, transplantation of Dll4 overexpressing EPCs increases the blood flow to the ischemic zone and improves cardiac function (129).

## Potential Insults Caused by Notch Inhibition to the Cardiovascular System

Considering the many roles of Notch in physiology and pathological states of the heart and of the vascular system, the risks of detrimental cardiovascular effects of Notch inhibition, especially in patients already affected by cardiovascular diseases, should be considered.

Inhibition of Notch signaling could have negative consequences on angiogenesis in two group of patients: (i) those with diabetes mellitus, which are characterized by impairment of neo-vascularization and wound healing, and (ii) those with coronary or peripheral atherosclerosis causing ischemia of the heart or other organs (130, 131), since they all rely on the development of collateral circulation to meet the oxygen needs. Furthermore, given the key role of Notch in the survival of cardiomyocytes, in the proliferation of CPCs and in the mobilization and functions of EPCs and MSCs, the possibility that Notch inhibition could interfere with myocardial repair or exacerbate pathological remodeling of an already damaged or pressure-overloaded myocardium should be considered.

**Figure 2:**
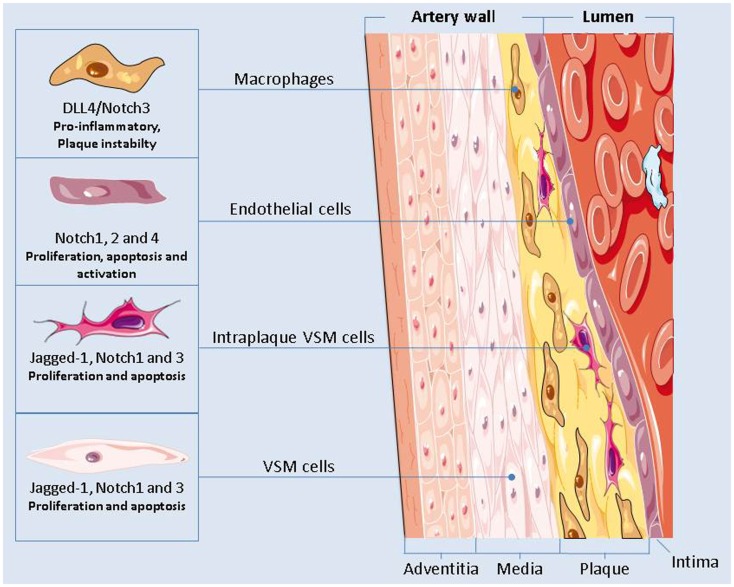
**The Notch signaling plays a major role in regulating the functions of the cells present in the vascular artery wall**. Notch inhibition could have an effect on the onset and progression of atherosclerosis by modulating the pro-inflammatory activity of macrophages, by causing endothelial cells dysfunctions, and by altering the apoptotic and proliferation rate of the vascular smooth muscle cells.

Notch inhibition could also worsen atherosclerosis by enhancing endothelial cells dysfunctions or by causing VSMCs apoptosis (Figure 2). Nevertheless, in macrophages activation of Dll4/Notch3 has been associated with plaque instability (Figure 2) (132) and inhibition of Dll4-mediated Notch signaling in metabolic syndrome has proven to be effective in slowing down the progression of atherosclerosis (133). Given the different roles played by Notch in the cellular elements of plaques, the consequences of Notch inhibition in atherosclerosis are still unclear and they should be further investigated.

The clinical studies conducted so far have shown no signs of cardiotoxicity associated with Notch inhibition for cancer therapy (47). One major side effect that has emerged from these trials is gastrointestinal toxicity and it has been found that intermittent dosing schedules of a Notch inhibitor can largely spare the gut, while maintaining anti-tumor efficacy (38). In addition, it has been found that administration of corticosteroids, which already are a component of some cancer regimens, may help ameliorate the gut toxicity of Notch inhibition (134).

Theoretical risks of long-term Notch inhibition have been postulated, such as damage to normal stem cells or increased incidence of certain cancers in which Notch acts as a tumor suppressor (47), and, similarly, these first trials may have missed long-term consequences of Notch inhibition on the cardiovascular system. Cardiotoxicity has been detected years after the last anthracycline dose in patients treated for childhood neoplasms (135) and risks of several cardiovascular disease has been found to be three to fivefold increased in 1474 survivors of Hodgkin lymphoma compared with the general population (136). The risk of anthracycline-induced cardiotoxicity is affected by gender and menopausal status (1) and in trastuzumab-treated cancer patients; cardiotoxicity is worsened by pre-existing cardiac pathologies (137). Since several clinical studies have found a high incidence of cardiovascular pathological conditions among the cancer patients, specific phase1 studies with Notch inhibitor including selected types of patients could uncover previously undetected cardiotoxicity. Additionally, as the clinical studies employing Notch inhibitors move toward combination treatment of Notch inhibitors with existing cancer drugs (38), the possibility of an additive/synergistic effect of the two drugs on cardiotoxicity should also be considered.

## Conclusive Remarks

As cancer progresses toward the status of chronic disease, new challenges arise and among them the possible damages of cancer treatments to the cardiovascular system. The Notch pathway has a tremendous potential as a new target in cancer therapy. For investigational Notch, as with other new anticancer agents, the interaction between cardiologists and oncologists will be crucial to design specific studies able to identify which patients could be at high risk of developing cardiotoxicity and to employ the best therapeutic strategy based on the assessment of the different risks.

## Conflict of Interest Statement

The authors declare that the research was conducted in the absence of any commercial or financial relationships that could be construed as a potential conflict of interest.

## Abbreviations

ADAM, a disintegrin and metalloprotease; AMI, acute myocardial infarction; AngII, angiotensin II; ApoE, apolipoprotein E; BM, bone marrow; CADASIL, cerebral autosomal-dominant arteriopathy with subcortical infarcts and leukoencephalopathy; cGMP, cyclic guanosine monophosphate; CHF, congestive heart failure; CPCs, cardiac progenitor cells; CREC, Cardiac Review and Evaluation Committee; CXCR4, chemokine (C–X–C motif) receptor 4; Dll4, delta-like ligand 4; ECD, endothelial dysfunction; eNOS, endothelial nitric oxide synthase; GSI, γ-secretase inhibitor; HER2, human epidermal growth factor receptor 2; HF, heart failure; HUVECs, human umbilical veins endothelial cells; ICAM-1, intracellular adhesion molecule-1; IL1β, interleukin 1β; LV, left ventricle; LVEF, left ventricle ejection fraction; MDR1, multi-drug resistant gene product 1; MSCs, mesenchymal stem cells; NF-κB, nuclear factor-kappa-light-chain-enhancer of activated B-cell; NIC, Notch intracellular; NO, nitric oxide; NT-proBNP, N-terminal of the pro-hormone brain natriuretic peptide; PEA3, polyomavirus enhancer activator 3; RBP-Jk, recombinant signal binding protein 1 for Jk; RT-3DE, three-dimensional echocardiography; ROS, reactive oxygen species; RV, right ventricle; Sca1, stem cell antigen 1; SDF-1, stromal derived factor 1; SOD, super oxide dismutase; T-ALL, T-cells acute lymphoblastic leukemia; T-DM1, trastuzumab (T) linked to the cytotoxic agent mertansine (DM1); TNFα, tumor necrosis factor α; VCAM-1, vascular cell adhesion molecule-1; VEGF, vascular endothelial growth factor; VSMCs, vascular smooth muscle cells.
